# Influence of Different Frying Processes on the Flavor Characteristics and Sensory Profile of Garlic Oil

**DOI:** 10.3390/molecules24244456

**Published:** 2019-12-05

**Authors:** Jie Sun, Baoguo Sun, Fazheng Ren, Haitao Chen, Ning Zhang, Yuyu Zhang

**Affiliations:** 1 Beijing Advanced Innovation Center for Food Nutrition and Human Health, College of Food Science & Nutritional Engineering, China Agricultural University, Beijing 100083, China; sunjeel@163.com (J.S.); sunbg@btbu.edu.cn (B.S.); renfazheng@cau.edu.cn (F.R.); 2 Key Laboratory of Functional Dairy, College of Food Science & Nutritional Engineering, China Agricultural University, Beijing 100083, China; 3 Beijing Laboratory for Food Quality and Safety, Beijing Technology & Business University, Beijing 100048, China; zh_ningts@btbu.edu.cn (N.Z.); zhangyuyu@btbu.edu.cn (Y.Z.)

**Keywords:** garlic oil, frying process, aroma compounds, sensory profiles, generating pattern, analysis

## Abstract

Fried garlic oil has been widely used in traditional Chinese cuisine and, recently, has become increasingly popular in food manufacturing. In this study, the effects of different initial and final frying temperature on the flavor characteristics and sensory profile of fried garlic oil were investigated using solvent-assisted flavor evaporation (SAFE) combined with gas chromatography–mass spectrometry (GC–MS). Results showed that the content of flavor compounds changed significantly as the frying temperature was increased. The sample that was treated at an initial temperature of 115 °C and a final temperature of 155 °C contained the highest amount of thioethers and heterocycles, mainly comprising dimethyl trisulfide, diallyl disulfide, and 2-vinyl-4*H*-1,2-dithiin. Partial least-squares regression elucidated the sensory attributes of fried and roasted garlic, showing a high correlation with thioethers and pyrazines. Furthermore, changes in the 2,6-dimethylpyrazine, dimethyl trisulfide, and diallyl disulfide concentrations were detected every 5 °C during the frying process (initial temperature, 115 °C; final temperature, 155 °C). Dimethyl trisulfide and diallyl disulfide concentrations showed irregular, downward trends, while 2,6-dimethylpyrazine concentration exhibited an increasing trend.

## 1. Introduction

Garlic (*Allium sativum* L.) is native to Western and Central Asia, and it has been cultivated and consumed extensively for centuries because of its medicinal and edible value [1]. The various biological activities of garlic extract include antioxidant, anticancer, antihypertensive, and age-resisting activities, and they have attracted increasing interest [2,3,4]. Additionally, processed garlic products in the domestic seasoning market, including fried garlic oil, have been applied in a variety of food products, such as chips, breads, and instant noodles, as a flavor ingredient. Accordingly, the use of fried garlic oil has been progressively increasing due to demand in the food manufacturing industry.

Frying is acknowledged as a common food preparation method and is widely used in industrial food production and domestic kitchens [5]. In this process, a small amount of raw garlic is soaked in heated vegetable oil for a short time to achieve a unique flavor. The fried garlic and oil are immediately separated once the garlic looks a golden appearance, and the oil is obtained and used as a flavoring oil. Complicated physical and chemical interactions, including oxidation, hydrolysis, and polymerization, occur in frying oil because of the oxygen, high temperature, and water released by the food, which produces flavor compounds [6,7]. Frying temperature is one of the important factors that affect the flavor and quality of fried products [8]. Overheating will cause external carbonization to produce a burnt smell, but the interior will be in a semi-ripe state. Hence, for such a process, frying conditions that contribute to the formation of flavor compounds should be adopted and optimized.

This frying process confers to fried garlic oil unique flavors with a slightly pungent taste as well as a salty odor. In contrast to numerous reports on the non-volatile compounds and health effects of garlic, little work has been performed to investigate the volatiles of garlic generated during the frying process. Volatile components of fried, oil-cooked, microwave-fried, baked, and microwave-baked garlic samples have been determined and compared by a modified simultaneous distillation extraction (SDE) apparatus [9]. Diallyl disulfide and diallyl trisulfide were found to be the dominant compounds in baked and microwave-baked garlic samples, while diallyl disulfide, methyl allyl disulfide, and vinyldithiins were the dominant compounds in fried, oil-cooked, and microwave-fried garlic samples, respectively [9]. Another study investigated the effect of drying techniques on the aroma quality of red garlic. Raw samples provided a higher amount of disulfides, mainly diallyl disulfide and allyl (*E*)-1-propenyl disulfide, whereas the the dried samples were characterized by an increase in trisulfides and cyclic sulfur compounds, such as thianes and thiolanes [10]. Again, using headspace gas analysis, dimethyl sulfide, allyl alcohol, diallyl sulfide, methyl allyl disulfide, and diallyl disulfide were identified as the major volatile compounds in stir-fried garlic [11]. These sulfur compounds were generated from thermal degradation of nonvolatile flavor precursors of garlic and thermal interactions of sugars, lipids, and nonvolatile flavor precursors of garlic [12]. It is obvious that most of the studies on volatiles present in garlic have been focused on the effect of process techniques. Attempts to evaluate the impact of process conditions on the aroma of garlic products are, however, are inadequate.

In our previous study, we investigated differences in the volatile flavor constituents of fresh garlic and fried garlic oil, in an attempt to illustrate a series of reactions potentially induced by the frying process and characteristic compounds of fried garlic oil, by analyzing odor activity values [13]. So far, investigations regarding the frying conditions and changes in key odorants of fried garlic oil during frying have not yet been reported.

Thus, the objective of this study was to determine effects of the frying process on the aroma and sensory profiles of garlic oil to optimize the process conditions (initial and final frying temperatures) using an induction cooker and to observe changes in key odorants by sensory analysis and SAFE-GC-MS analysis. The results will provide a guide for selecting appropriate frying processes to produce high-quality garlic oil.

## 2. Results

### 2.1. Effects of Different Initial and Terminational Temperatures on Volatile Flavor Compounds

The different initial and final frying temperatures strongly influenced the volatile flavor compounds of garlic oil. The corresponding results are shown in Table 1 and Table 2, respectively. At different initial temperatures, a total of 44 compounds were detected in the garlic samples, including two alcohols, fifteen esters, seven aldehydes, and twenty heterocyclic compounds (Table 1). The total volatile content in 115Sam was 52.785 mg/g, which was significantly higher than that of other samples. Thus, the effects of different final temperatures on flavor compounds were investigated when temperature was constant at 115 °C, which was chosen as the optimal initial frying temperature. The results are shown in Table 2, and a total of 48 volatile compounds were identified in all samples.

**Figure 1:**
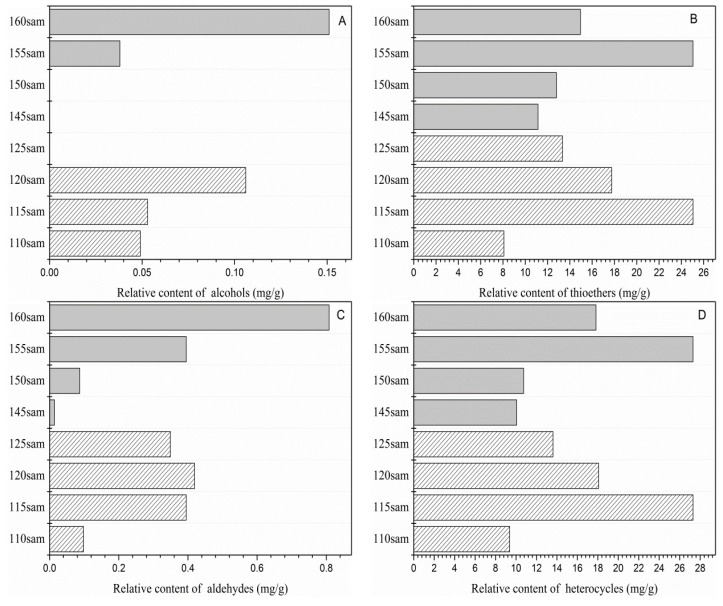
Total content of flavor compounds at different initial and final temperatures. (**A**) Alcohols. (**B**) Thioethers. (**C**) Aldehydes. (**D**) Heterocycles.

Garlic oil samples contained a large number of volatile flavor compounds; the most abundant were thioethers and heterocyclic compounds, which constituted the structural components of the aroma of garlic oil. Volatile thioether compounds contributed significantly to the characteristic aroma of fried garlic oil. Figure 1B demonstrates the effects of frying temperatures on the thioethers.

As the initial frying temperature ranged from 110 to 125 °C, an initial increase in the quantity of thioethers was observed, and then, their contents decreased. The same trend occurred at the final temperature. The heterocyclic compounds detected included sulfur-, nitrogen-, and oxygen-containing compounds. The contents of heterocyclic compounds among initial temperature samples were significantly different, while no marked difference was observed in 145 and 150Sam (Figure 1D). 2-Vinyl-4H-1,2-dithiin and 3-vinyl-4H-1,2-dithiin, which had a pungent scent, greatly affected the total content of heterocyclic compounds. 115Sam and 155Sam contained the highest content of vinyl dithiins, which was about 40% higher than in other samples. Pyrazines, such as 2,5-dimethyl-3-ethylpyrazine and 2-methyl-pyrazine, were the main nitrogen compounds in the fried garlic oil and had previously been identified in roasted garlic [14]. In addition, oxygen-containing heterocyclic compounds were the products of the Maillard reaction, including 2,3-dihydro-3,5-dihydroxy-6-methyl-4(H)-pyran-4-one and furaneol. Furaneol was only present in 115Sam, 155 Sam, and 160Sam at a low concentration, while the frying treatment caused a notable change in 2,3-dihydro-3,5-dihydroxy-6-methyl-4(H)-pyran-4-one. With an increase in the final temperature, the content and types of aldehydes exhibited an upward trend (Figure 1C), indicating that a high final temperature promoted thermal oxidative degradation of fats. With respect to the initial temperature samples, 110Sam exhibited a lower content than 115Sam, 120Sam, and 125Sam. The total contents of the two alcohols found in different garlic oil samples are shown in Figure 1A. Significant differences in the alcohol contents were observed between the samples at initial and final temperatures. No alcohols were detected in 125Sam, 145Sam, and 150Sam, which contrasted with previous studies [12], where oil-treated garlic samples were found to produce a significant amount of 2-propen-1-ol.

### 2.2. Relationship between Sensory Descriptors and Flavor Compounds

Sensory evaluation was performed using descriptive sensory analysis. The mean intensity values of the eight kinds of garlic oil samples are shown in Table 3 and Table 4. Different initial temperatures had significant effects on the salty, fried, roasted, and vegetable-like attributes (*p* < 0.05). Nevertheless, the notes of salty, roasted, and raw garlic showed significant differences at different final temperatures (*p* < 0.05). Additionally, 115Sam and 155Sam showed the highest salty and fried attributes among the samples. The garlic oil samples exhibited similar sour intensities, which were all scored lower than 4.

**Figure 2:**
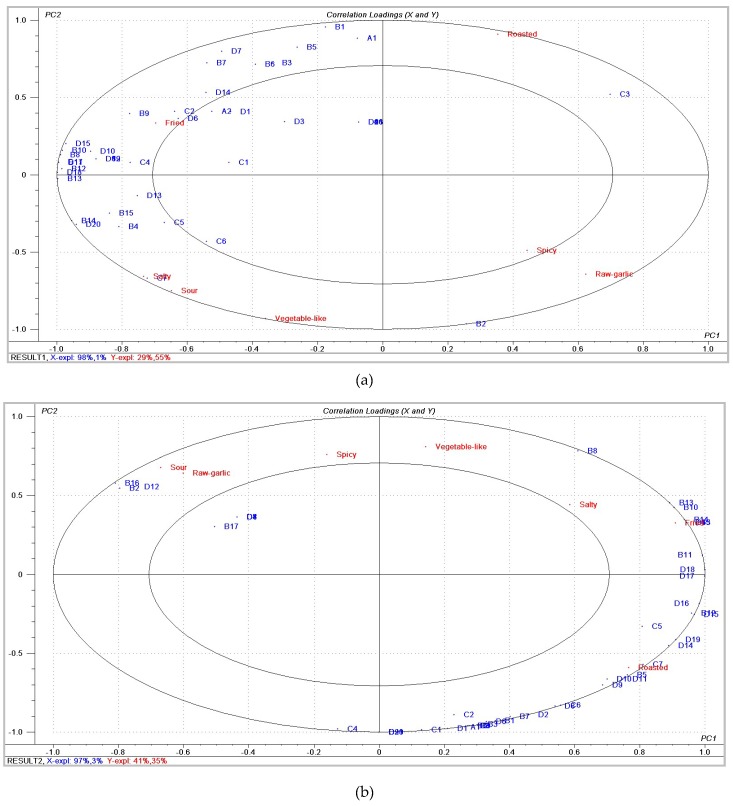
(**a**) Correlation loading plot between the seven sensory attributes and volatile compounds at different initial temperatures. (**b**) Correlation loading plot between the seven sensory attributes and volatile compounds at different final temperatures.

PLSR was performed to determine the relationship between sensory attributes and volatile compounds. The PLSR model of different initial temperatures between the matrices of volatile compounds and sensory attributes provided a two-factor model explaining 99% of the variance in X (volatile compounds) and 84% of that in Y (sensory attributes), as shown in Figure 2a. In addition, the PLSR model of different final temperatures provided a two-factor model explaining 100% of the variance in X (volatile compounds) and 76% of that in Y (sensory attributes), as shown in Figure 2b. Figure 2a,b present correlation loading plots, in which the small ellipse indicated 50% of the explained variance, and the large ellipse was a unit circle indicating 100% of the explained variance [15]. Aroma compounds inside the inner ellipse were poorly connected with sensory attributes, while those located between the two ellipses were considered to be correlated with sensory attributes.

As shown in Figure 2a, the fried attribute was closely related to diallyl disulfide, 4-heptenal, and 2,6-dimethylpyrazine, which was in agreement with the analysis results (Table 1), where the 115Sam exhibited a higher content of these compounds. The salty attribute was located in the same quadrant as sour and vegetable-like attributes. The salty and sour attributes showed strong correlations with (*E*,*E*)-2,4-decadienal, while the vegetable-like attribute showed little correlation with any flavor compounds. The raw garlic and spicy attributes were related to 1-allyloxy-2,3-epoxypropane. As shown in Figure 2b, the fried attribute was strongly correlated with (*E*)-1-allyl-2-(prop-1-en-1-yl)disulfane, diallyl trisulfide, (*E*)-allyl(prop-1-en-1-yl)sulfane, 1,2-di((*E*)-prop-1-en-1-yl)disulfane, and 2,6-dimethylpyrazine. The roasted attribute exhibited positive correlations with (*E*)-1-methyl-2-(prop-1-en-1-yl)disulfane, (*E*,*E*)-2,4-decadienal, 2,5-dimethyl-3-ethylpyrazine, and 3,5-dimethyl-2-ethylpyrazine. On the left side of PC1 (principal component 1), the raw garlic and sour attributes were noted to be significantly correlated with 1-allyloxy-2,3-epoxypropane, (*Z*)-allyl(prop-1-en-1-yl)sulfane, and 3-ethyl-pyridine. The salty attribute exhibited a correlation with 1-allyl-2-isopropyldisulfane. Vegetable-like and spicy attributes showed little correlation with any flavor compounds.

### 2.3. Generating the Patterns of 2,6-dimethylpyrazine, Dimethyl Trisulfide, and Diallyl Disulfide in Garlic Oil

PLSR analysis showed that thioethers and pyrazines contributed greatly to the flavor characteristics of garlic oil. Furthermore, the flavor compound contents were highest when the initial temperature was 115 °C and the final temperature was 155 °C. Under these conditions, changes in the concentrations of 2,6-dimethylpyrazine, dimethyl trisulfide, and diallyl disulfide were detected every 5 °C by GC–MS (Table 5).

**Figure 3:**
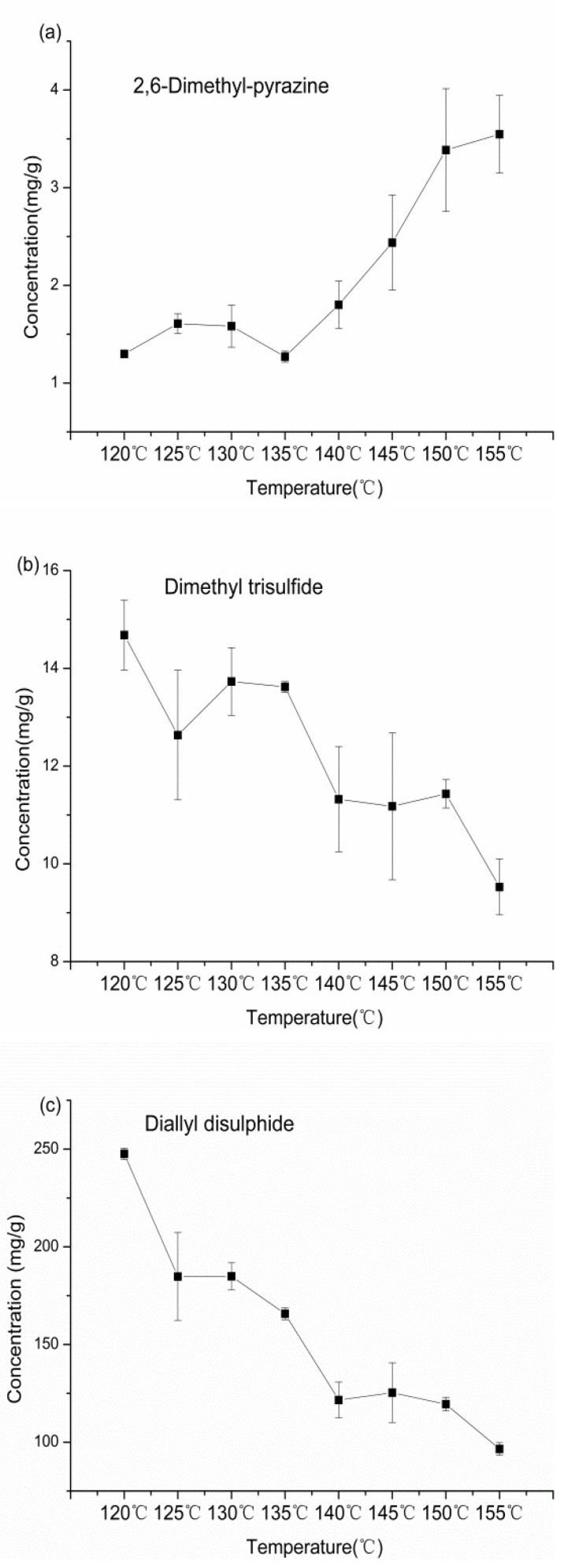
Changes in concentrations of 2,6-Dimethyl-pyrazine (**a**), Dimethyl trisulfide (**b**), and Diallyl disulfide (**c**) in garlic oil at different cut-off temperatures.

As shown in Figure 3a, the concentration of 2,6-dimethylpyrazine was in the range of 1.27–3.55 mg/g. In the early stage of frying (120–135 °C), the content slightly increased at first and then decreased, followed by a rapid increase from 135 °C to 155 °C. Variations in the dimethyl trisulfide concentration at different temperatures are shown in Figure 3b. The maximum amount of dimethyl trisulfide was reached at 120 °C, resulting from the decomposition of a large amount of allicin. Subsequently, the amount decreased at an irregular rate. With respect to diallyl disulfide, the changes in concentrations (Figure 3c) were similar to those of dimethyl trisulfide, which reduced quickly during the early stage of frying.

## 3. Discussion

Results of the GC–MS analysis on flavor compounds showed that the aroma profile of garlic oil was clearly related to the frying process. The main class of thioethers observed was disulfides, which accounted for over 50% of the total content of thioethers and dominated the profile of garlic oil. This result was in accordance with a previous study [9]. Diallyl sulfides and polysulfides can be generated from the thermal decomposition of allicin or by alliinase action on a mixture of alliin and cystine [16]. However, as a high temperature and long processing time were used in the manufacture of heated garlic, alliinase was inactivated [17,18,19]. Therefore, increasing the frying temperature and time lowered the disulfide contents. Since it took the longest time for 110Sam to reach the same final temperature in the frying process, 110Sam exhibited a markedly lower content.

In all samples, 2-vinyl-4*H*-1,2-dithiin exhibited the highest content, followed by 3-vinyl-4*H*-1,2-dithiin. This indicated that these components were the major degradation product of allicin during frying. Vinyl dithiins have been reported as isomeric cyclic organosulfides. and are major components in distilled garlic oil [20,21]. 2-Vinyl-4*H*-1,2-dithiin is the major vinyl dithiin, while 3-vinyl-4*H*-1,2-dithiin is a minor component, in agreement with our results. Vinyl dithiins might be derived from the thermal degradation of allicin or the interaction of degradation products of nonvolatile flavor precursors in garlic [22]. Furthermore, allicin, when extracted by nonpolar organic solvents (mostly hexane) at slightly elevated temperatures, has been reported to transform into vinyl dithiins—as major components [23].

Despite the comparably lower concentration of pyrazines, because of their low odor threshold, the contribution to the flavor profile of garlic oil could not be ignored. The content of pyrazines showed an upward trend as final temperatures increased from 145 to 160 °C. The Maillard reaction is regarded as a major mechanism for pyrazine formation. Its products have been reported in model systems containing alliin or deoxyalliin with glucose or inosine-5′-monophosphate. Nonvolatile flavor precursors in garlic, such as -glutamylalk(en)ylcystein and alk(en)yl-cysteine *S*-oxides, are considered to contribute significantly to the formation of pyrazines identified in fried or baked garlic [12].

Aldehydes are the dominant odor compounds in cooked products because of their relatively low odor thresholds, which were probably generated from thermal interactions of lipids and nonvolatile flavor precursors in garlic, eventually becoming part of the overall flavor of fried garlic oil. In previous studies, acetaldehyde was identified as a volatile component of heat-treated garlic [12]. However, it was not found at all in our study. Its absence could be explained by different extraction methods and its low concentration in garlic oil.

2-Propen-1-ol was generated by thermal degradation of alliin and its biochemical precursor deoxyalliin [24]. Yu et al. [9] proposed a [2,3]-sigmatropic rearrangement mechanism for 2-propen-1-ol formation from alliin. Furthermore, as noted by Rizzi [25], 2-propen-1-ol might react with allylthio radicals, allyl mercaptan, or alliin/deoxyalliin to give diallyl sulfide. This assumption supported a decrease in the amount of 2-propen-1-ol with prolonged heating and explains why low 2-propen-1-ol contents were detected in the present study. However, Molina-Calle et al. [26] found no 2-propen-1-ol when studying the volatile profile of black garlic using headspace-GC–MS. Therefore, different heat treatment methods have a significant influence on 2-propen-1-ol formation.

In order to evaluate the differences in the sensory quality of garlic oil from different frying processes, they were subjected to PLSR between sensory descriptors and flavor compounds. For initial temperatures, diallyl disulfide, 4-heptenal, 2,6-dimethylpyrazine, (*E*,*E*)-2,4-deca-dienal, and 1-allyloxy-2,3-epoxypropane were positively correlated with sensory descriptors. (*E*,*E*)-2,4-decadienal contributed to the salty and sour notes and was consistent with 125Sam, which had high scores in salty and sour notes and a high content of (*E*,*E*)-2,4-decadienal. The presence of 1-allyloxy-2,3-epoxypropane, exhibiting raw garlic and spicy attributes, in 125Sam explained that a difference in flavor existed compared to the other three varieties. On the other hand, the content of these substances positively correlated with sensory descriptors in 155Sam oil, which was four times that in 160Sam oil; thus, the sensory evaluation of the two oils was differed greatly, and the sensory evaluation score of 155Sam oil was the highest. Therefore, the sensory profile of garlic oil could be enhanced by improving the content of thioethers and pyrazines.

The concentration curve of 2,6-dimethylpyrazine indicated that increasing the cut-off temperature markedly improved the Maillard reaction rate. Variation in the dimethyl trisulfide concentration increased and then decreased. It increased when the oil temperature reached 130 °C because it was also generated by the thermal degradation of amino acids, but there was an overall downward trend. This result was in agreement with the previous report, which showed that the amount of dimethyl trisulfide increased under heating for short periods and decreased with extended heating [27]. Diallyl disulfide showed the same trend, indicating that the two components were unstable at high temperatures and gradually decomposed with increasing oil temperature. The results obtained above showed that controlling the initial and final temperatures could produce garlic oil with different flavor profiles.

## 4. Materials and Methods

### 4.1. Chemicals

Dichloromethane and sodium sulfate were purchased from Sinopharm Chemical Reagent Co., Ltd. (Beijing, China). Dichloromethane was freshly distilled before experiments. Normal alkanes (C_10_–C_28_) and internal standard tetradecane were purchased from Sigma-Aldrich (Shanghai, China) and were of at least analytical grade. High-purity 2,6-dimethylpyrazine (95%), dimethyl trisulfide (95%), and diallyl disulfide (95%) were also purchased from Sigma-Aldrich (Shanghai, China) and used for quantitation.

### 4.2. Sample Preparation

Fresh white garlic cloves were purchased from Jinxiang (34.52 °S, 116.7 °E) located in Jining city, Shandong province, China, in May 2018. The outer skin of the garlic cloves was manually separated and peeled. Soybean oil was used and produced in China.

Garlic cloves were automatically ground into a paste by a multifunction cooking machine. The frying procedure was adapted from Zhang et al. [28] with some modifications. A stainless-steel wok 20 cm in diameter was used for frying. Soybean oil (300 g) was first added into the wok and heated to 115 °C using an induction cooker at an output power of 500 W. The temperature was measured with an oil thermometer. Garlic paste (300 g) was then added and fried until the oil reached the designed final temperature (145 °C, 150 °C, 155 °C, and 160 °C). Immediately, the temperature of the garlic paste–oil mixture dropped first, caused by the moisture, and then increased during the frying process. Heating was immediately stopped when the mixture reached the designed final temperature. The garlic paste was then removed, and the garlic oil was cooled to room temperature using an ice-water bath and used for further analysis.

Initial soybean oil frying temperatures of 110 °C (110Sam), 115 °C (115Sam), 120 °C (120Sam), and 125 °C (125Sam) were firstly examined to obtain the optimal initial frying temperature. Then final frying temperatures of 145 °C (145Sam), 150 °C (150Sam), 155 °C (155Sam), and 160 °C (160Sam) were examined under the optimal initial frying temperature. All temperatures were designed according to industrial production. The frying processes were repeated three times under the same conditions, and the average results were taken into consideration. All garlic oils were sealed in storage bottles and stored at −20 °C until analysis was carried out.

### 4.3. Solvent-Assisted Flavor Evaporation (SAFE)

Garlic oil (50 g) was extracted with dichloromethane (100 mL) by shaking at room temperature for 1 h (Grant OLS200, Cambridgeshire, UK). The combined extracts were then subjected to high-vacuum distillation using SAFE. The resulting distillate was dried over anhydrous sodium sulfate and filtered. The final distillate was concentrated to 1 mL using a Vigreux column.

### 4.4. Gas Chromatography–Mass Spectrometry (GC–MS)

GC–MS was performed using a Thermo Fisher Trace 1310 gas chromatograph (Thermo Fisher Scientific, Waltham, MA, USA) coupled with a Thermo Fisher mass spectrometer (Thermo Fisher Scientific, Waltham, MA, USA). Isolates were analyzed using TG-Wax MS columns (30 m × 0.25 mm i.d., 0.25 μm, Thermo Fisher Scientific, Waltham, MA, USA). The carrier gas was high-purity helium (99.999%) at a fixed flow rate of 1.0 mL/min into the column. The initial oven temperature was 40 °C for 1 min, which was then increased to 130 °C at a rate of 2 °C/min and held for 1 min, and finally, increased to 220 °C at a rate of 10 °C/min and held for 8 min. The sample was injected in 1 μL at 250 °C in a 1:30 split ratio.The mass detector conditions were as follows: ionization energy, 70 eV; ion source temperature, 250 °C; mass range, *m/z* 45–500; solvent delay, 5 min.

Volatile flavor compounds were identified by comparing their retention indices and mass spectra with those of reference standards in the NIST 14 mass spectra database. Retention indices were calculated for each volatile compound using the retention times of a homologous series of *n*-alkanes (C_10_–C_28_).

### 4.5. Quantitative Analysis

The relative quantities of the volatile flavor compounds were mainly quantitated using an internal standard method. The concentrate was spiked with tetradecane (internal standard, 1.5256 mg/mL in dichloromethane) and calculated by comparing the peak area with that of tetradecane. Among them, 2,6-dimethylpyrazine, dimethyl trisulfide, and diallyl disulfide were separately measured by constructing standard curves. The final results were the averages of three replicates.

### 4.6. Sensory Evaluation

A descriptive sensory evaluation was conducted by 10 experienced panelists (five males and five females) from Beijing Key Laboratory of Flavor Chemistry. The evaluation was performed in a sensory room with single cubicles at a controlled temperature (25 °C). The samples (10 g of fried garlic oil) were placed in separate 30 mL bottles fitted with airtight PTFE-lined screw tops before analysis and coded with a 2-digit number randomly. Descriptors were developed with reference to the literature [29]. Panelists were recruited for training sessions. Then they smelled the samples and discussed the odor attributes. The seven descriptors were agreed upon by the panelists in the training, namely, salty, fried, roasted, vegetable-like, spicy, sour, and raw garlic. A descriptive sensory analysis was performed by a scoring linein with a scale from 0 (not detected) to 9 (very intense odor). The average of each odor descriptor was calculated and shown in Table 3 and Table 4.

### 4.7. Statistical Analysis

The experimental results were expressed as means ± standard deviation. The attribute intensity scores obtained by sensory descriptive analysis were submitted to one-way analysis of variance (ANOVA) using Duncan’s multiple range test in SPSS 19.0 software (IBM Corporation, Chicago, IL, USA). Significant differences were defined as *p* < 0.05. Partial least-squares regression (PLSR) was used to explore relationships among volatile flavor compounds and sensory descriptors using Unscrambler version 9.7 software (CAMO ASA, Oslo, Norway). Origin version 8.5 software (Origin Lab Inc., Northampton, MA, USA) was used to process GC–MS data.

## 5. Conclusions

The effects of using different initial and final temperatures on the flavor characteristics and sensory profiles of garlic oil were investigated. The total amount of flavor compounds in 115Sam and 155Sam were significantly higher than that in the other samples, which was mainly reflected in the increased content of thioether and heterocyclic compounds. The content of flavor compounds in samples varied as follows: 115Sam showed the highest content (52.785 mg/g) of heterocycles, followed by 120Sam (36.36 mg/g), 125Sam (27.319 mg/g), and 110Sam (17.612 mg/g), and 155Sam showed the highest content (52.785 mg/g) of heterocycles, followed by 160Sam (33.7331 mg/g), 150Sam (23.62 mg/g), and 145Sam (21.209 mg/g). The fried, salty, and roasted attributes strongly correlated with the levels of thioether and pyrazine compounds, such as diallyl disulfide, 2,6-dimethylpyrazine, (*E*)-1-methyl-2-(prop-1-en-1-yl)disulfane, diallyl trisulfide, and (*E*,*E*)-2,4-decadienal, which contributed greatly to the flavor characteristics of garlic oil. To reveal the flavor changes, selected 2,6-dimethylpyrazine, dimethyl trisulfide, and diallyl disulfide concentration trends were measured every 5 °C during the frying process, with an initial temperature of 115 °C and final temperature of 155 °C. Importantly, dimethyl trisulfide and diallyl disulfide showed similar trends, with the concentration decreasing in an irregular fashion as the cut-off temperature increased. In contrast, the 2,6-dimethylpyrazine concentration exhibited a clear upward trend. Therefore, the flavor characteristics of garlic oil can be changed by controlling the frying temperature. The following research will focus on the changes and formation mechanisms of other flavor substances.

## Figures and Tables

**Table 1:** Volatile flavor compounds of fried garlic oil at different initial temperatures.

Number	Compounds ^a^	Concentration (mg/g) ^b^				RI	Identification Method ^c^
		110 °C	115 °C	120 °C	125 °C		
Alcohols							
A1	2-Propen-1-ol	0.049 ± 0.008 ^a^	0.038 ± 0.021 ^a^	0.075 ± 0.05 ^b^	—	1123	MS/RI
A2	(*E*)-3-Penten-2-ol	—	0.015 ± 0.011 ^a^	0.031 ± 0.019 ^b^	—	1179	MS/RI
Thioethers							
B1	Dimethyl disulfide	0.021 ± 0.001 ^a^	0.019 ± 0.011 ^a^	0.026 ± 0.02 ^a^	—	1075	MS/RI
B2	1-Allyloxy-2,3-epoxypropane	—	—	—	0.026 ± 0.018	1123	MS
B3	Diallyl sulfide	0.059 ± 0.006 ^a^	0.073 ± 0.052a	0.115 ± 0.078 ^b^	0.024 ± 0.016 ^c^	1147	MS/RI
B4	(*E*)-Allyl(prop-1-en-1-yl)sulfane	—	0.023 ± 0.009 ^a^	—	0.01 ± 0.002 ^b^	1188	MS
B5	(*E*)-1-Methyl-2-(prop-1-en-1-yl)disulfane	0.101 ± 0.016 ^a^	0.107 ± 0.067 ^a^	0.157 ± 0.098 ^a,b^	0.038 ± 0.018 ^a,c^	1206	MS
B6	Methyl allyl disulfide	0.246 ± 0.006 ^a^	0.315 ± 0.189 ^a^	0.445 ± 0.27 ^a,b^	0.133 ± 0.06 ^a,c^	1278	MS/RI
B7	Dimethyl trisulfide	0.178 ± 0.008 ^a^	0.25 ± 0.134 ^b^	0.293 ± 0.163 ^b^	0.098 ± 0.048 ^c^	1369	MS/RI
B8	1-Allyl-2-isopropyldisulfane	0.015 ± 0.003 ^a^	0.042 ± 0.012 ^b,c^	0.03 ± 0.011 ^b^	0.02 ± 0.001 ^a,d^	1421	MS
B9	Diallyl disulfide	3.149 ± 0.055 ^a^	9.334 ± 2.569 ^b^	6.475 ± 1.543 ^c,d^	4.538 ± 0.416 ^a,d^	1468	MS/RI
B10	(*E*)-1-Allyl-2-(prop-1-en-1-yl)disulfane	1.059 ± 0.117 ^a^	3.219 ± 0.809 ^b,c^	2.305 ± 0.588 ^b^	1.415 ± 0.056 ^a,d^	1457	MS
B11	Allyl *n*-propyl sulfide	—	0.021 ± 0.014 ^a^	0.024 ± 0.018 ^a^	—	1450	MS
B12	Methyl allyl trisulfide	1.727 ± 0.096 ^a^	4.59 ± 0.419 ^b^	3.577 ± 0.554 ^c^	2.586 ± 0.13 ^d^	1574	MS/RI
B13	1,2-Di-((*E*)-prop-1-en-1-yl)disulfane	0.027 ± 0.001 ^a^	0.098 ± 0.006 ^b^	0.058 ± 0.011 ^c^	0.047 ± 0.001 ^c^	1741	MS
B14	Diallyl trisulfide	1.502 ± 0.188 ^a^	6.926 ± 0.353 ^b^	4.252 ± 1.646 ^c^	4.409 ± 0.635 ^c^	1771	MS/RI
B15	(*Z*)-1-Allyl-3-(prop-1-en-1-yl)trisulfane	—	0.023 ± 0.005 ^a^	—	0.008 ± 0.001 ^b^	1776	MS
Aldehydes							
C1	Hexanal	—	0.022 ± 0.018 ^a^	0.048 ± 0.037 ^b^	0.016 ± 0.016 ^a^	1085	MS/RI
C2	4-Heptenal	—	0.026 ± 0.014 ^a^	0.041 ± 0.028 ^b^	—	1169	MS/RI
C3	(*E*)-2-Heptenal	0.029 ± 0.009	—	—	—	1321	MS/RI
C4	(*Z*)-2-Heptenal	—	0.04 ± 0.019 ^a^	0.049 ± 0.027 ^a^	0.018 ± 0.007 ^b^	1322	MS/RI
C5	(*E*)-2-Octenal	—	0.011 ± 0.004 ^a^	0.014 ± 0.002 ^a^	0.01 ± 0.002 ^b^	1425	MS/RI
C6	(*E,E*)-2,4-Heptadienal	0.069 ± 0.004a	0.134 ± 0.022 ^b^	0.163 ± 0.012 ^b^	0.146 ± 0.019 ^b^	1465	MS/RI
C7	(*E,E*)-2,4-Decadienal	—	0.162 ± 0.019 ^a^	0.104 ± 0.04 ^b^	0.159 ± 0.001 ^a^	1759	MS/RI
Heterocyclic compounds							
D1	2-Pentylfuran	—	0.01 ± 0.005 ^a^	0.024 ± 0.002 ^b^	—	1233	MS/RI
D2	Methylpyrazine	—	—	0.046 ± 0.03	—	1266	MS/RI
D3	4-Methylpyrimidine	0.025 ± 0.003 ^a^	0.032 ± 0.012 ^a^	—	0.006 ± 0.002 ^b^	1267	MS
D4	2-Methyl-2-thiazolidine	—	—	0.014 ± 0.001	—	1295	MS/RI
D5	2-Ethenylthiophene	—	0.019 ± 0.011	—	—	1296	MS/RI
D6	2,6-Dimethylpyrazine	0.041 ± 0.001 ^a^	0.082 ± 0.03 ^b^	0.108 ± 0.028 ^b^	0.045 ± 0.009 ^a^	1319	MS/RI
D7	2,5-Dimethylpyrazine	0.044 ± 0.008 ^a^	0.061 ± 0.033 ^a,b^	0.071 ± 0.033 ^b^	0.014 ± 0.002 ^c^	1356	MS/RI
D8	4-Ethylpyridine	—	0.006 ± 0.001	—	—	1356	MS/RI
D9	3-Ethylpyridine	—	—	0.014 ± 0.006	—	1374	MS/RI
D10	2-Ethyl-6-methylpyrazine	0.072 ± 0.003 ^a^	0.187 ± 0.043 ^b^	0.178 ± 0.017 ^b^	0.104 ± 0.013 ^c^	1381	MS/RI
D11	3-Methyl-2-ethylpyrazine	—	—	0.045 ± 0.019	—	1397	MS/RI
D12	3,5-Dimethyl-2-ethylpyrazine	—	0.006 ± 0.001	—	—	1428	MS/RI
D13	2,5-Dimethyl-3-ethylpyrazine	0.044 ± 0.001 ^a^	0.126 ± 0.009 ^b^	0.143 ± 0.017 ^b^	0.1 ± 0.008 ^c^	1440	MS/RI
D14	3H-1,2-Dithiole	0.047 ± 0.008 ^a^	0.073 ± 0.043 ^a^	0.097 ± 0.031 ^a,b^	0.038 ± 0.018 ^a,c^	1510	MS/RI
D15	3-Methyl-3*H*-1,2-dithiole	0.485 ± 0.025 ^a^	1.29 ± 0.264 ^b^	0.791 ± 0.178 ^a^	0.523 ± 0.079 ^a^	1570	MS
D16	2-Furanmethanol	—	—	0.051 ± 0.012	—	1670	MS/RI
D17	3-Vinyl-4*H*-1,2-dithiin	2.868 ± 0.031 ^a^	8.074 ± 0.731 ^b^	5.476 ± 0.751 ^c^	3.958 ± 0.131 ^d^	1711	MS/RI
D18	2-Vinyl-4*H*-1,2-dithiin	5.646 ± 0.956 ^a^	16.572 ± 1.891 ^b^	10.591 ± 2.486 ^c,d^	8.398 ± 2.045 ^a,d^	1821	MS/RI
D19	Furaneol	—	0.095 ± 0.011	—	—	2045	MS/RI
D20	2,3-Dihydro-3,5-dihydroxy-6-methyl-4(*H*)-pyran-4-one	0.109 ± 0.005 ^a^	0.664 ± 0.082 ^b^	0.429 ± 0.165 ^c,d^	0.432 ± 0.142 ^b,d^	2282	MS/RI

**Table 2:** Volatile flavor compounds of fried garlic oil at different final temperatures.

Number	Compounds ^a^	Concentration (mg/g) ^b^				RI	Identification Method ^c^
		145 °C	150 °C	155 °C	160 °C		
Alcohols							
A1	2-Propen-1-ol	—	—	0.038 ± 0.021 ^a^	0.151 ± 0.033 ^b^	1123	MS/RI
Thioethers							
B1	Dimethyl disulfide	—	0.005 ± 0.004 ^a^	0.019 ± 0.011 ^b^	0.049 ± 0.003 ^c^	1075	MS/RI
B2	1-Allyloxy-2,3-epoxypropane	0.036 ± 0.007 ^a^	0.024 ± 0.012 ^a^	—	—	1122	MS
B3	Diallyl sulfide	0.031 ± 0.001 ^a^	0.039 ± 0.005 ^a^	0.073 ± 0.052 ^b^	0.16 ± 0.001 ^c^	1147	MS/RI
B4	(*E*)-Allyl(prop-1-en-1-yl)sulfane	—	—	0.023 ± 0.009	—	1188	MS
B5	(*E*)-1-Methyl-2-(prop-1-en-1-yl)disulfane	0.032 ± 0.004 ^a^	0.045 ± 0.014 ^a^	0.107 ± 0.067 ^b^	0.116 ± 0.003 ^b^	1284	MS
B6	Methyl allyl disulfide	0.179 ± 0.015 ^a^	0.206 ± 0.033 ^a^	0.315 ± 0.189 ^a^	0.642 ± 0.023 ^b^	1277	MS/RI
B7	Dimethyl trisulfide	0.12 ± 0.015 ^a^	0.164 ± 0.048 ^a^	0.25 ± 0.134 ^a^	0.411 ± 0.001 ^b^	1368	MS/RI
B8	1-Allyl-2-isopropyldisulfane	0.022 ± 0.003 ^a^	0.028 ± 0.009 ^a^	0.042 ± 0.012 ^b^	0.01 ± 0.001 ^c^	1420	MS
B9	Allyl *n*-propyl sulfide	—	—	0.021 ± 0.014 ^a^	0.074 ± 0.003 ^b^	1450	MS
B10	(*E*)-1-Allyl-2-(prop-1-en-1-yl)disulfane	1.06 ± 0.201 ^a^	1.24 ± 0.357 ^a^	3.219 ± 0.809 ^b^	0.918 ± 0.065 ^a^	1456	MS
B11	Diallyl disulfide	4.94 ± 0.649 ^a^	5.378 ± 1.634 ^a^	9.334 ± 2.569 ^b^	6.065 ± 0.082 ^a^	1473	MS/RI
B12	Methyl allyl trisulfide	1.905 ± 0.504 ^a^	2.427 ± 0.853 ^a^	4.59 ± 0.419 ^b^	3.571 ± 0.179 ^b^	1574	MS/RI
B13	1,2-Di-((*E*)-prop-1-en-1-yl)disulfane	0.038 ± 0.007 ^a^	0.043 ± 0.012 ^a^	0.098 ± 0.006 ^b^	0.032 ± 0.005 ^a^	1741	MS
B14	Diallyl trisulfide	2.763 ± 0.929 ^a^	3.186 ± 0.966 ^a^	6.926 ± 0.353 ^b^	2.907 ± 0.117 ^a^	1770	MS/RI
B15	(*Z*)-1-Allyl-3-(prop-1-en-1-yl)trisulfane	—	—	0.023 ± 0.005	—	2282	MS/RI
B16	(*Z*)-Allyl(prop-1-en-1-yl)sulfane	0.009 ± 0.002 ^a^	0.01 ± 0.006 ^a^	—	—	1187	MS
B17	1-Allyl-3-propyltrisulfane	0.003 ± 0.001	—	—	—	1707	MS/RI
Aldehydes							
C1	Hexanal	—	0.012 ± 0.004 ^a^	0.022 ± 0.018 ^a^	0.128 ± 0.007 ^b^	1085	MS/RI
C2	4-Heptenal	—	0.026 ± 0.002 ^a^	0.026 ± 0.014 ^a^	0.069 ± 0.004 ^b^	1169	MS/RI
C3	(*Z*)-2-Heptenal	—	—	0.04 ± 0.019 ^a^	0.143 ± 0.008 ^b^	1322	MS/RI
C4	Nonanal	0.014 ± 0.007 ^a^	—	—	0.059 ± 0.01 ^b^	1391	MS/RI
C5	(*E*)-2-Octenal	—	0.006 ± 0.001 ^a^	0.011 ± 0.004 ^b^	0.009 ± 0.013 ^b^	1425	MS/RI
C6	(*E,E*)-2,4-Heptadienal	—	—	0.134 ± 0.022 ^a^	0.232 ± 0.006 ^b^	1465	MS/RI
C7	(*E,E*)-2,4-Decadienal	—	0.031 ± 0.003 ^a^	0.162 ± 0.019 ^b^	0.163 ± 0.009 ^b^	1798	MS/RI
C8	(*E*)-2-Heptenal	—	0.012 ± 0.005	—	—	1321	MS/RI
C9	Heptanal	—	—	—	0.005 ± 0.001	1187	MS/RI
Heterocyclic compounds							
D1	2-Pentylfuran	—	—	0.01 ± 0.005 ^a^	0.047 ± 0.013 ^b^	1233	MS/RI
D2	4-Methylpyrimidine	—	—	0.032 ± 0.012 ^a^	0.069 ± 0.003 ^b^	1267	MS
D3	3-Methylpyridine	—	—	—	0.01 ± 0.001	1217	MS
D4	2-Methyl-2-thiazolidine	—	0.005 ± 0.001	—	—	1294	MS/RI
D5	2-Ethenylthiophene	—	—	0.019 ± 0.011	—	1296	MS/RI
D6	2,5-Dimethylpyrazine	0.018 ± 0.002 ^a^	0.041 ± 0.009 ^a^	0.082 ± 0.03 ^b^	0.19 ± 0.007 ^c^	1319	MS/RI
D7	3-Methyl-2-ethylpyrazine	—	0.012 ± 0.013	—	—	1402	MS
D8	4-Ethypyridine	—	—	0.006 ± 0.001	—	1356	MS/RI
D9	6-Methyl-2-ethyl-pyrazine	0.039 ± 0.001 ^a^	0.081 ± 0.012 ^b^	0.187 ± 0.043 ^c^	0.227 ± 0.027^ac^	1380	MS/RI
D10	2,5-Dimethyl-3-ethylpyrazine	0.053 ± 0.004 ^a^	0.08 ± 0.018 ^a^	0.126 ± 0.009 ^b^	0.141 ± 0.011 ^b^	1440	MS/RI
D11	3,5-Dimethyl-2-ethylpyrazine	—	—	0.006 ± 0.001 ^a^	0.007 ± 0.003 ^a^	1428	MS/RI
D12	3-Ethylpyridine	0.01 ± 0.001 ^a^	0.017 ± 0.008 ^a^	—	—	1374	MS/RI
D13	2,6-Dimethylpyrazine	0.01 ± 0.006 ^a^	0.02 ± 0.007 ^a^	0.061 ± 0.033 ^b^	0.098 ± 0.015 ^c^	1326	MS/RI
D14	3*H*-1,2-Dithiole	0.021 ± 0.003 ^a^	0.027 ± 0.019 ^a^	0.073 ± 0.043 ^b^	0.064 ± 0.011 ^b^	1510	MS/RI
D15	3-Methyl-3*H*-1,2-dithiole	0.491 ± 0.105 ^a^	0.557 ± 0.199 ^a^	1.29 ± 0.264 ^b^	0.969 ± 0.03 ^b^	1570	MS
D16	3-Vinyl-4*H*-1,2-dithiin	2.674 ± 0.564 ^a^	3.019 ± 1.116 ^a^	8.074 ± 0.731 ^b^	5.515 ± 0.918 ^c^	1711	MS/RI
D17	2-Vinyl-4*H*-1,2-dithiin	6.626 ± 1.858 ^a^	6.733 ± 2.29 ^a^	16.572 ± 1.891 ^b^	10.043 ± 0.525 ^c^	1822	MS/RI
D18	2,3-Dihydro-3,5-dihydroxy-6-methyl-4*H*-pyran-4-one	0.115 ± 0.046 ^a^	0.146 ± 0.095 ^a^	0.664 ± 0.082 ^b^	0.293 ± 0.001 ^c^	2281	MS/RI
D19	Furaneol	—	—	0.095 ± 0.011 ^a^	0.072 ± 0.014 ^b^	2045	MS/RI
D20	3,4-Dihydro-2*H*-pyran	—	—	—	0.015 ± 0.001	1133	MS
D21	2-Ethyltetrahydrothiophene	—	—	—	0.059 ± 0.004	1494	MS

^a^ Compounds were identified in this condition. ^b^ Values are shown as the mean ± SD (standard deviation) of replicates in each sample. ^c^ Identification based on Nist 14 mass spectral database; published retention indices. ^d^ Different letters in the same row indicate significant differences according to Duncan’s test (*p* < 0.05).

**Table 3:** Mean intensity values of the seven attributes at different initial temperatures ^a^.

Sample	Salty	Fried	Roasted	Vegetable-Like	Spicy	Sour	Raw Garlic
110Sam	5.00 a	6.00 ab	6.14 a	3.86 a	6.14	1.86	5.14
115Sam	7.00 bc	7.57 a	4.14 bc	5.43 ab	5.64	2.29	4.00
120Sam	6.29 c	5.29 b	4.57 b	4.71 a	4.86	2.00	3.57
125Sam	6.93 c	5.14 b	2.71 c	6.43 b	6.29	2.29	5.86
*p*	0.025	0.025	0.010	0.032	0.388	0.892	0.066

**Table 4:** Mean intensity values of the seven attributes at different final temperatures ^a^.

Sample	Salty	Fried	Roasted	Vegetable-Like	Spicy	Sour	Raw Garlic
145Sam	4.67 a	5.83	4.00 a	5.00	5.33	3.00	5.67 a
150Sam	6.67 bc	6.17	4.83 a	6.00	6.00	3.33	4.50 b
155Sam	7.00 b	6.83	6.50 b	5.67	5.50	2.50	3.83 bc
160Sam	5.17 ac	6.00	6.67 b	4.50	5.00	2.33	3.33 c
*p*	0.013	0.623	0.01	0.21	0.778	0.656	0

^a^ Mean scores for each attribute; the different letters denote that the values are significantly different (*p* < 0.05) by use of Duncan’s multiple comparison tests.

**Table 5:** Concentrations of three compounds detected at different cut-off temperatures.

Compounds	Calibration Equations (Y*10^^6^)	R^2^	Concentration (mg/g)							
			120 °C	125 °C	130 °C	135 °C	140 °C	145 °C	150 °C	155 °C
Dimethyl trisulfide	y = 2.625 x − 3.113	0.999	14.68 ± 0.72	12.63 ± 1.32	13.73 ± 0.69	13.62 ± 0.11	11.32 ± 1.08	11.18 ± 1.5	11.43 ± 0.29	9.53 ± 0.57
Diallyl disulfide	y = 1.893 x + 42.220	0.997	247.6 ± 2.88	184.8 ± 22.47	184.9 ± 6.95	165.75 ± 3.03	121.6 ± 9.17	125.35 ± 15.3	119.46 ± 3.52	96.54 ± 3.31
2,6-Dimethyl-pyrazine	y = 1.945 x + 36.005	0.999	1.3 ± 0.01	1.61 ± 0.10	1.4 ± 0.26	1.27 ± 0.06	1.8 ± 0.24	2.44 ± 0.49	3.39 ± 0.63	3.55 ± 0.40
